# Disturbed acid-base transport: an emerging cause of hypertension

**DOI:** 10.3389/fphys.2013.00388

**Published:** 2013-12-24

**Authors:** Ebbe Boedtkjer, Christian Aalkjaer

**Affiliations:** Department of Biomedicine, Aarhus UniversityAarhus, Denmark

**Keywords:** blood pressure, SLC4, SLC9, SLC26, intracellular pH, acidosis, alkalosis

## Abstract

Genome-wide association studies and physiological investigations have linked alterations in acid-base transporters to hypertension. Accordingly, Na^+^-coupled HCO^−^_3_-transporters, Na^+^/H^+^-exchangers, and anion-exchangers have emerged as putative mechanistic components in blood pressure disturbances. Even though hypertension has been studied extensively over the last several decades, the cause of the high blood pressure has in most cases not been identified. Renal, cardiovascular, and neuronal dysfunctions all seem to play a role in hypertension development but their relative importance and mutual interdependency are still being debated. Multiple functional and structural alterations have been described in patients and animals with hypertension but it is typically unclear whether they are causes or consequences of hypertension or represent mechanistically unrelated associations. Perturbed blood pressure regulation has been demonstrated in several animal models with disrupted expression of acid-base transporters; and reciprocally, disturbed acid-base transport function has been described in hypertensive individuals. In addition to regulating intracellular and extracellular pH, Na^+^-coupled HCO^−^_3_-transport, Na^+^/H^+^-exchange, and anion-exchange also contribute to water and electrolyte balance in cells and systemically. Since acid-base transporters are widely expressed, alterations in transport activities likely affect multiple cell and organ functions, and it is a significant challenge to determine the mechanisms linking perturbed acid-base transport function to hypertension. It is the purpose of this review to evaluate the current evidence for involvement of acid-base transporters in hypertension development and discuss the cellular and integrative mechanisms, which may link changes in acid-base transport to blood pressure disturbances.

## Introduction

Around 40% of the adult population has hypertension (WHO, 2013), and although the cause of the high blood pressure is typically unknown, it is well established that hypertension is a leading cause of e.g., ischemic heart disease, stroke, and renal disease. Consequently, hypertension has detrimental effects on human health and severe socioeconomic consequences.

Blood pressure control takes place through a complex interplay between multiple organ systems. The mean perfusion pressure of the systemic circulation is determined by the product of the cardiac output and the peripheral vascular resistance; and consequently, the blood pressure depends on e.g., the cardiac contractile function, the circulating blood volume, the vascular smooth muscle and endothelial function in resistance arteries, the vascular structure, the viscosity of the blood, the secretory function of endocrine glands, and the sympathetic nervous tone. In line with the complex nature of blood pressure regulation, multiple mechanisms of deregulation have been proposed; and as described by Page's mosaic theory, essential hypertension in most cases is likely to be a multifactorial disease involving several organ systems in a complex interplay (Page, 1949). In some cases, however, these diverse changes may have a common denominator such as changes in oxidative stress or immune cell function (Harrison, 2013). Frequently described causes for hypertension involve changes in the kidneys, the resistance vasculature, and the autonomic nervous system. As discussed in further detail below, the function of these organs all depends on appropriate levels of membrane acid-base transport activity. In congruence, over the last few decades, several experimental investigations and genetic association studies have proposed that alterations in the expression and function of acid-base transporters can be involved in blood pressure deregulation (Table 1). For the most part, however, the mechanistic background linking disturbed acid-base transport activity to hypertension is unknown, and it is the scope of the current review to summarize and discuss possible pathophysiological mechanisms.

**Table 1:** **Genetic and experimental associations between acid-base transporters and blood pressure**.

**Gene name**	**SNP/locus/modification**	**Phenotype**	**References**
*SLC4A1*	rs5036	Hypertension	Kokubo et al., 2006
	rs2857078	Preeclampsia	Morrison et al., 2010
*SLC4A2*	rs2303934	Hypertension	Sõber et al., 2009
*SLC4A4*	SNP	Hypertension	Yang et al., 2012
*SLC4A5*	rs7571842	Salt-sensitive hypertension	Barkley et al., 2004; Hunt et al., 2006; Taylor et al., 2009, 2012, 2013; Carey et al., 2012
	rs10177833		
	rs8179526		
	hcv1137534		
	Knockout	Hypertension	Gröger et al., 2012
*SLC4A7*	rs13082711	Hypertension	Ehret et al., 2011
	Knockout	Resting hypertension but protected against hypertension to Ang-II and L-NAME	Boedtkjer et al., 2011
*SLC9A1*	Knockout	Hypotension	Boedtkjer et al., 2012
	Knockout	Protection against pulmonary hypertension	Yu et al., 2008
*SLC9A2*	SNP No. 4 (intron 6)	Hypertension	Iwai et al., 2006
*SLC9A3*	rs4957061	Preeclampsia	Morrison et al., 2010
	Knockout	Hypotension	Schultheis et al., 1998; Noonan et al., 2005
*SLC26A4*	Knockout	Protection against mineralocorticoid-induced hypertension	Verlander et al., 2003
	Overexpression	Cl^−^-sensitive hypertension	Jacques et al., 2013
*SLC26A6*	Knockout	Protected against fructose-induced hypertension	Singh et al., 2008
*SLC26A9*	Knockout	Hypertension	Amlal et al., 2013

### Genetic association studies

Multiple associations between genetic polymorphisms in acid-base transporters and disturbed blood pressure regulation have been described (Table 1). These studies propose that anion-exchangers (AE1, AE2), Na^+^,HCO^−^_3_-cotransporters (NBCe1, NBCe2, NBCn1) and Na^+^/H^+^-exchangers (NHE2, NHE3) can be involved in hypertension development. It should be noted that for several of the single nucleotide polymorphisms (SNPs), which have been shown to correlate with hypertension in some studies, other studies have not been able to confirm the association. This may in part be due to differences in the investigated population sizes but may also reflect differences in e.g., ethnicities, gender, age or co-morbidity—which considering the complexity of blood pressure regulation may modify the effects of specific gene variants. Depending on the location of the SNPs, it is possible that they can alter the function or the expression of the transporters, but in many circumstances the SNPs described to associate with hypertension may not themselves be causative but rather be in linkage disequilibrium with functionally important SNPs residing within the coding region or in important gene regulatory elements.

While the observed genetic associations do not themselves provide mechanistic insight, they are important in elucidating new targets to be studied experimentally. In some cases, experimental investigations support the link between the acid-base transporters and hypertension and provide evidence for a pathophysiological mechanism. The importance of NBCn1 for blood pressure regulation was for instance proposed by a recent genome-wide association study linking the rs13082711 SNP located 10 kb 5′ of the coding region of *SLC4A7* to hypertension (Ehret et al., 2011), and this finding was concurrently reinforced by an experimental study showing that NBCn1 knockout mice have a complex blood pressure phenotype and an altered vascular function (Boedtkjer et al., 2011), see section The Resistance Arteries and Microvasculature. Likewise, multiple studies linking SNPs in *SLC4A5* to salt-sensitive hypertension (Barkley et al., 2004; Hunt et al., 2006; Taylor et al., 2009, 2012, 2013; Carey et al., 2012) have been supported by the finding that knockout mice for NBCe2 are hypertensive (Gröger et al., 2012), see section The Kidneys.

### The kidneys

The kidneys have classically been considered the primary organs for long-term regulation of blood pressure (Guyton, 1991). The proposed mechanism of renal blood pressure regulation involves a feedback system where an excess arterial pressure causes the kidneys to excrete more salt and water, decreasing the circulating blood volume, and ultimately reducing the blood pressure (Guyton, 1991). The relationship between arterial pressures and urinary salt output has been suggested to be exceptionally steep and the system has been proposed to have infinite feedback gain (Guyton, 1991), i.e., it is able to fully compensate any deviation in blood pressure away from the set-point. This dominating role of the kidneys for blood pressure regulation has more recently been investigated in a series of elegant experiments using transgenic mice. Focusing specifically on the impact of the renin angiotensin system, reciprocal cross-transplantations of fully vascularized kidney transplants between AT1A receptor deficient and wild type mice were performed to determine the relative importance of AT1A receptors expressed in renal (e.g., the renal epithelium, the renal vasculature or other renal components) and extra-renal (e.g., the resistance vasculature, the CNS or the adrenals) tissues for baseline blood pressure and angiotensin II induced hypertension. These studies support that the hypertensive effects of angiotensin II are predominantly dependent on renal AT1A receptors (Crowley et al., 2006). In contrast, the reduced resting blood pressure observed in AT1A deficient mice was found to depend approximately equally on renal and extra-renal AT1A receptors (Crowley et al., 2005). Although subsequent experiments have shown that selective inactivation of signaling pathways in the vascular wall can protect against hypertension development (see section The Resistance Arteries and Microvasculature), these findings support that the kidneys play a prominent—although not exclusive—role for blood pressure regulation and hypertension development.

As described above, the kidneys contribute to blood pressure regulation by modulating body water and electrolyte homeostasis through the control of urinary excretion. Simplistically, enhanced Na^+^ and water retention by the kidneys is expected to increase the circulating blood volume, increase the venous return to the heart—and through the Frank-Starling mechanism—to raise the stroke volume, cardiac output, and blood pressure. However, while an increased cardiac output has been reported in the early phases of essential hypertension, cardiac output has been described to be normal in patients with sustained essential hypertension (Lund-Johansen, 1991). Thus, there seems to be a shift in hypertensive patients from an early phase with a hyperkinetic circulation (i.e., increased cardiac output) to an established phase with an increased total peripheral resistance and a normal cardiac output. The importance of an increased peripheral resistance for hypertension induced by salt retention has recently been supported by the finding that smooth muscle specific abrogation of the myosin light chain kinase (He et al., 2011) or the G-protein coupled pathways required for activation of the rho-kinase (Wirth et al., 2008) attenuates the development of deoxycorticosterone acetate (DOCA)-salt hypertension. Although the mechanisms linking disturbed renal salt and water handling and increased circulating blood volume to an increased peripheral vascular resistance remain controversial, it is well-documented that renal Na^+^ retention leads to hypertension. Acid-base transporters may play a very significant role both in mediating renal Na^+^ retention (see below) and in modifying the subsequent increase in peripheral vascular resistance (see section The Resistance Arteries and Microvasculature).

Acid-base transporters are expressed in all segments of the nephrons and collecting ducts; and since the actions of many acid-base transporters involve co- or counter-transport of Na^+^ and/or Cl^−^, they can directly influence renal NaCl handling. A detailed description of the expression patterns and functional roles of acid-base transporters in the different renal tubular segments is outside the scope of the current review but the potential role of Na^+^-coupled acid-base transporters for blood pressure regulation is exemplified by the Na^+^/H^+^-exchanger NHE3 (Slc9a3): this transporter—which is expressed in renal proximal tubules, some thin descending limbs, and in the thick ascending limbs of the loop of Henle—has been shown to play a major role for renal Na^+^ reabsorption, and in congruence, under low to normal NaCl intake, NHE3 knockout mice are hypotensive (Schultheis et al., 1998; Noonan et al., 2005). Similarly, transport of acid-base equivalents coupled to Cl^−^ has been convincingly shown to modify blood pressure: knockout of the anion-exchanger pendrin (Slc26a4), which contributes to Cl^−^ reabsorption in the distal nephron (Wall et al., 2004), has been shown to protect against mineralocorticoid-induced hypertension (Verlander et al., 2003) while overexpression of pendrin in the intercalated cells was recently shown to cause hypertension (Jacques et al., 2013). It should be noted that both direct effects of increased or reduced acid-base transporter activity as well as compensatory effects of altered delivery of acid-base, Na^+^ or Cl^−^ loads to downstream tubule segments may contribute to changes in electrolyte homeostasis and blood pressure deregulation. Although further experimental evidence is required to test this hypothesis, it has for instance been suggested that knockout of NBCe2—which is expressed in the renal collecting ducts (Damkier et al., 2007; Gröger et al., 2012)—causes hypertension because of a compensatory increase in NBCn1 transport activity (Gröger et al., 2012). NBCn1 is expressed in the renal medullary collecting ducts (Boedtkjer et al., 2008) and due to the 1:1 Na^+^ to HCO^−^_3_stoichiometry of NBCn1 rather than the 1:2 stoichiometry of NBCe2, the putative shift from NBCe2- to NBCn1-mediated HCO^−^_3_ reabsorption could increase the concomitant Na^+^ reabsorption and contribute to the raised blood pressure (Gröger et al., 2012). Consistent with the involvement of renal Na^+^ retention in the hypertensive phenotype of NBCe2 deficient mice, two of the *SLC4A5* SNPs associated with hypertension in humans were also found to associate with salt-sensitivity of the raised blood pressure (Carey et al., 2012).

In addition to direct effects of the Na^+^- or Cl^−^-coupled acid-base transporters on renal salt retention, changes in intracellular acid-base balance in the renal epithelium are also likely to affect other transport systems: numerous ion channels [e.g., the ROMK K^+^-channel (Wang et al., 1990; Fakler et al., 1996; Choe et al., 1997)] and transporters [e.g., the Na^+^/K^+^-ATPase (Russell et al., 1983; Eaton et al., 1984; Breitwieser et al., 1987)] are modulated by the local pH and may therefore, contribute to salt retention or wasting as a consequence of disturbed intracellular pH control.

Consistent with an association between acid-base transporters and blood pressure regulation, altered renal expression levels and acid-base transport function have been reported in hypertension: Na^+^/H^+^-exchange activity has been shown to be increased in brush border membranes of proximal tubules from spontaneously hypertensive compared to Wistar-Kyoto rats (Morduchowicz et al., 1989; Gesek and Schoolwerth, 1991; Dagher and Sauterey, 1992; Hayashi et al., 1997). Whether this functional difference is explained by an increased expression of NHE3 in spontaneously hypertensive rats is controversial (Hayashi et al., 1997; Sonalker et al., 2004) but the increased Na^+^/H^+^-exchange activity may also be related to a lower expression of the regulatory protein NHERF1 (Kobayashi et al., 2004). Na^+^,HCO^−^_3_-cotransport has also been found to be altered in hypertensive animals; however, the increased NBCe1 expression reported in the renal cortex of spontaneously hypertensive compared to Wistar-Kyoto rats (Sonalker et al., 2004) was not confirmed in experiments using immortalized proximal tubular epithelial cells, which showed lower NBCe1 expression and Na^+^,HCO^−^_3_-cotransport activity in cells from spontaneously hypertensive compared to Wistar-Kyoto rats (Pedrosa et al., 2007). The expression of both NBCe1 and NHE3 has been found to be increased in the renal cortex of rats with noradrenaline-induced hypertension (Sonalker et al., 2008). Although it is currently unclear whether these changes in acid-base transporter expression and function are causative, compensatory or unrelated to the blood pressure disturbances, they warrant further experimental study to elucidate their mechanistic relevance.

### The resistance arteries and microvasculature

The resistance vasculature plays a central role in regulating peripheral vascular resistance and blood pressure in the short term, but it has been debated whether primary changes in the vascular wall can be responsible for sustained blood pressure disturbances: based on the suggestion by Guyton that the renal regulation of fluid balance has infinite gain, it has been argued that a blood pressure rise in response to vasoconstriction will elicit an enhanced renal water and salt excretion, which will ultimately normalize blood pressure (Guyton, 1990). As a consequence, long-term blood pressure disturbances should be possible only if renal fluid control is affected either directly or in response to changes in renal artery function (Guyton, 1990). Supporting a role for the vasculature in long-term regulation of blood pressure, pharmacological agents (e.g., Ca^2+^ channel blockers) thought to act primarily on the vascular smooth muscle cells have sustained blood pressure lowering effects (Fleckenstein et al., 1985); and over recent years, genetic modifications specific to cells of the vascular wall have been shown to modify blood pressure. Smooth muscle specific inactivation of Arhgef1 or G_12_-G_13_-LARG, important for rho-kinase signaling, was for instance shown to protect against hypertension development during chronic angiotensin II infusion and DOCA-salt treatment (Wirth et al., 2008; Guilluy et al., 2010) while smooth muscle specific deletion of the NO-activated soluble guanylyl cyclase (Groneberg et al., 2010) or endothelium-specific expression of a dominant negative peroxisome proliferator-activated receptor (PPAR)-γ (Pelham et al., 2013) was found to cause hypertension. Also, smooth muscle specific deletion of Dicer (Albinsson et al., 2011), which is required for microRNA processing, or of the Na^+^/Ca^2+^-exchanger (Zhang et al., 2010) was shown to cause hypotension. Although the ability of vasoactive drugs and vascular genetic modifications to change blood pressure in the long term may be explained by actions on the renal arteries and therefore, ultimately depend on altered renal fluid handling, these findings demonstrate that primary modifications of vascular function can alter blood pressure and that these effects are not fully corrected by compensatory changes in renal excretory function.

The impact of extracellular acid-base disturbances on the function of resistance arteries has long been appreciated; and while the mechanistic background is not comprehensively understood, extracellular acid-base disturbances are assumed to contribute substantially to the metabolic regulation of blood flow. As a consequence of extracellular acid-base disturbances or as independent disorders, the intracellular acid-base balance can be altered in a number of disease conditions and probably as a result of genetic polymorphisms or mutations in acid-base transporters. As recently reviewed in detail (Boedtkjer and Aalkjaer, 2012), intracellular acid-base disturbances have major effects on contractile and relaxant functions of resistance arteries. Three transport systems dedicated to movement of acid-base equivalents have been described in the plasma membrane of vascular smooth muscle and endothelial cells: acid extrusion is mediated by the electroneutral Na^+^,HCO^−^_3_-cotransporter NBCn1 (Aalkjaer and Hughes, 1991; Boedtkjer et al., 2006, 2011; Thomsen et al., 2013) and the Na^+^/H^+^-exchanger NHE1 (Boedtkjer and Aalkjaer, 2009; Boedtkjer et al., 2012), while base extrusion is mediated by Cl^−^/HCO^−^_3_-exchange (Aalkjaer and Hughes, 1991; Boedtkjer et al., 2013). Whereas both NBCn1 and NHE1 contribute to cellular acid extrusion during intracellular acidification, NBCn1 is the primary acid extrusion mechanism important for control of resting steady-state intracellular pH in both vascular smooth muscle and endothelial cells (Boedtkjer et al., 2011, 2012). Hence, knockout of NHE1 affects steady-state intracellular pH only in the absence of CO_2_/HCO^−^_3_ (Boedtkjer et al., 2012).

We recently showed that knockout of NBCn1 has severe consequences for blood pressure regulation in mice: NBCn1 knockout mice are moderately hypertensive at rest (~10 mmHg) but show reduced blood pressure increases to NO-synthase inhibition and angiotensin II infusion (Boedtkjer et al., 2011). Association between genetic polymorphisms in NBCn1 and human hypertension has also been demonstrated (Ehret et al., 2011), see section Genetic Association Studies. Consistent with the changes in blood pressure regulation, we showed that sustained intracellular acidification of endothelial cells, whether induced by knockout of NBCn1 (in the presence of CO_2_/HCO^−^_3_) or NHE1 (in the absence of CO_2_/HCO^−^_3_), inhibits basal and acetylcholine-stimulated NO synthesis and vasorelaxation (Boedtkjer et al., 2011, 2012; Thomsen et al., 2013). In addition, sustained acidification of vascular smooth muscle cells induced by similar approaches inhibits rho-kinase-dependent Ca^2+^-sensitivity and attenuates myogenic responsiveness and vasoconstriction to noradrenaline (Boedtkjer et al., 2011, 2012; Thomsen et al., 2013). The combination of anti-contractile and anti-relaxant effects of low intracellular pH in the vascular wall likely explains the complex blood pressure phenotype of the NBCn1 knockout mice, which depending on the activity in the affected signaling pathways under certain conditions display higher and under other conditions lower blood pressure than wild type mice (Boedtkjer et al., 2011). Adding to the complexity, the anti-contractile effect of low intracellular pH in the vascular smooth muscle cells is evident during sustained (i.e., lasting several minutes to hours) intracellular acidification (Boedtkjer et al., 2006, 2011, 2012), whereas acute (i.e., taking place within a few tens of seconds) intracellular acidification causes abrupt vasoconstriction (Aalkjaer and Mulvany, 1988) probably due to competition between H^+^ and Ca^2+^ for buffer binding (Abercrombie and Hart, 1986; Batlle et al., 1993) and as a result of changes in cellular Ca^2+^ handling (Nielsen et al., 1991; Kozak et al., 2005; Restini and Bendhack, 2006). Under both physiological and pathological conditions, sustained changes in intracellular pH and associated anti-contractile effects are likely to predominate *in vivo*. Since contraction of vascular smooth muscle cells is associated with an increased intracellular acid load (Aalkjaer and Mulvany, 1991), it appears that a major role of membrane acid-base transport in these cells is to maintain intracellular pH at a sufficiently alkaline level that the Ca^2+^ sensitivity is not reduced. This may be particularly important during sustained contractions when the Ca^2+^-sensitivity of the vascular smooth muscle cells is usually increased (Boedtkjer et al., 2006). Supporting the importance of defending the intracellular pH of vascular smooth muscle cells during contractions, we have recently found that NBCn1 is activated in a calcineurin-dependent manner by the raised intracellular [Ca^2+^] during depolarization or agonist-stimulation (Danielsen et al., 2013). In addition to the vasomotor effects of intracellular acidification, we have found that intracellular alkalinization of endothelial cells—induced by inhibition or reversal of anion-exchange activity—impedes vasorelaxation by reducing intercellular coupling and endothelium-dependent hyperpolarizations (Boedtkjer et al., 2013). Although additional investigations are required to fully determine the pathophysiological significance of altered acid-base balance in the vascular wall, the recent evidence highlighted above strongly supports that pH-induced changes in artery function should be considered a possible mechanistic component in cardiovascular disease.

Besides changes in artery function, it should be appreciated that changes in the structure of the vascular wall may contribute to blood pressure disturbances (Folkow et al., 1958; Sivertsson, 1970; Aalkjaer et al., 1987b). Increased media thickness in the resistance arteries is a characteristic finding in essential hypertension and carries independent prognostic value (Rizzoni et al., 2003; Mathiassen et al., 2007). Furthermore, changes in arterial media thickness has been reported in models of disturbed acid-base transport (Yu et al., 2008; Boedtkjer et al., 2012), but its putative mechanistic role for blood pressure deregulation is not comprehensively understood (Boedtkjer and Aalkjaer, 2013). Further evidence is required to determine the role of intracellular pH regulation for structural adaptation of the vascular wall but it should be noted that numerous studies on non-vascular cell types have demonstrated that acid-base transport particularly by NHE1 modifies cell proliferation and migration (Pedersen, 2006; Schwab et al., 2012), which are critical processes underlying structural plasticity. In addition to structural changes in the resistance arteries, the disappearance of capillaries and pre-capillary arterioles (a phenomenon known as rarefaction) is important for the development of hypertension (Humar et al., 2009) and could play a role in blood pressure disturbances induced by altered acid-base transport. Although endothelial dysfunction has been shown to be associated with micro-vascular rarefaction (Humar et al., 2009), the potential effects of intracellular acid-base disturbances in the vessel wall on angiogenesis and vessel remodeling need further investigation.

In additional support of a pathogenic role of acid-base transporters for hypertension development, evidence has been provided that Na^+^/H^+^-exchange activity is increased in vascular smooth muscle cells of arteries from spontaneously hypertensive rats compared to Wistar-Kyoto rats (Izzard and Heagerty, 1989; Scuteri et al., 1995; Orlov et al., 2000). Noteworthy, however, steady-state intracellular pH did not differ between the vascular smooth muscle cells from adult hypertensive and normotensive rats under physiological conditions, i.e., in the presence of CO_2_/HCO^−^_3_ (Izzard and Heagerty, 1989; Scuteri et al., 1995). Conversely, a difference in steady-state intracellular pH in the presence of CO_2_/HCO^−^_3_ was reported between young (5 weeks old) spontaneously hypertensive and Wistar-Kyoto rats, which may suggest that the effect of NHE1 over-activity is temporally variable and could be involved at an early critical time point when the blood pressure disturbance and abnormal artery structure develop (Izzard and Heagerty, 1989). Alternatively, it is possible that NHE1 affects the vascular structure through transport-independent mechanisms or changes in pH of local restricted spaces with no discernible effect on global intracellular pH, as has been demonstrated in other cell types (Ro and Carson, 2004; Stock and Schwab, 2006). It is also possible that the vascular effects of NHE1 are secondary to changes in the function of other organ systems. The finding that NHE1 may act independently of global intracellular pH in the vascular smooth muscle cells is consistent with our previous data showing that arteries isolated from NHE1 knockout mice have reduced media thickness compared to arteries isolated from wild type mice despite no appreciable difference in steady-state intracellular pH in the presence of CO_2_/HCO^−^_3_ (Boedtkjer et al., 2012).

### The central and autonomic nervous systems

The regulation of sympathetic activity takes place through the cardiovascular center in the brain stem, which integrates multiple afferent inputs (e.g., from baroreceptors, chemoreceptors, and areas within the CNS) to control the autonomic output to e.g., the heart, kidneys, adrenals, and the resistance vasculature. The importance of afferent and efferent autonomic innervation for blood pressure control is underscored by the findings that renal denervation (Symplicity HTN-1 Investigators, 2011) or electric stimulation of the baroreceptors (Heusser et al., 2010) are promising treatment modalities for hypertensive patients not responding to conventional therapy.

Resistance arteries and veins in some vascular beds [e.g., the mesenteric (Birch et al., 2008)] are subject to intense sympathetic innervation while other vascular beds are without appreciable innervation and controlled primarily by local mechanical and metabolic factors. Increased sympathetic innervation of arteries has been reported in hypertensive rats (Mangiarua and Lee, 1990; Kunes et al., 1991), and it has been demonstrated that hypertension in spontaneously hypertensive rats can be prevented by reducing the sympathetic innervation (Brock et al., 1996). In humans, micro-neurography has been used to assess the sympathetic nervous activity, and an increased sympathetic nervous drive has been demonstrated in patients with essential hypertension and even in young (~24 years old) individuals with borderline hypertension (Anderson et al., 1989). In experiments where spill-over of noradrenaline from sympathetic nerves was assessed, enhanced net-release of noradrenaline was found in patients with essential hypertension (Esler et al., 1981). In small arteries isolated from humans with essential hypertension, indirect evidence has been provided for an increased uptake of noradrenaline into nerve endings in the vascular wall, both in untreated individuals with essential hypertension (Aalkjaer et al., 1987b), in persons genetically predisposed for essential hypertension (Aalkjaer et al., 1987a), and in patients already receiving antihypertensive treatment (Aalkjaer et al., 1989). Although force development of isolated small arteries to stimulation of the intramural nerves has been shown to correlate negatively with blood pressure in a normotensive population (Nielsen et al., 1992), the current evidence points to sympathetic vasoconstriction as a contributor to the elevated peripheral resistance in essential hypertension and proposes that changes in sympathetic function is an early pathogenic change, which precedes the development of established essential hypertension.

Regulation of intracellular pH in the peripheral autonomic nervous system has not been investigated in any major detail but seems to have both HCO^−^_3_-dependent and -independent components: in cultured sympathetic neurons, Na^+^/H^+^-exchange activity was suggested as the dominating acid extrusion mechanism, though HCO^−^_3_-dependent acid extrusion was also found to enhance intracellular pH recovery after an intracellular acid load and to increase steady-state intracellular pH (Tolkovsky and Richards, 1987). Na^+^/H^+^-exchange is also a prominent mechanism of cellular acid extrusion in the central nervous system (Chesler and Nicholson, 1985). In addition, multiple Na^+^-dependent HCO^−^_3_-transporters contribute to intracellular pH regulation in both neurons and glial cells: the Na^+^-driven Cl^−^/HCO^−^_3_-exchanger NDCBE was originally cloned from human brain (Grichtchenko et al., 2001); and functionally, Na^+^-dependent Cl^−^/HCO^−^_3_-exchange has been shown to be prominent in both invertebrate neuronal tissue (Boron and De Weer, 1976; Russell and Boron, 1976; Thomas, 1976) and mammalian neurons and glia (Schwiening and Boron, 1994; Shrode and Putnam, 1994). Also the electroneutral Na^+^,HCO^−^_3_-cotransporter NBCn1 (Boedtkjer et al., 2008) and NCBE/NBCn2 (Chen et al., 2008) have been shown to be widely expressed in the cerebral and cerebellar cortex and in multiple subcortical regions. Consistent with high expression levels in the hippocampus (Boedtkjer et al., 2008), NBCn1 has been functionally demonstrated in cultured hippocampal neurons (Cooper et al., 2005). Furthermore, the electrogenic Na^+^,HCO^−^_3_-cotransporter NBCe1 has been found to be prominently expressed in rat and mouse brain (Rickmann et al., 2007; Majumdar et al., 2008) and functionally demonstrated in both neurons (Svichar et al., 2011) and glia (Deitmer and Schlue, 1989). With respect to base extrusion, transcripts for multiple anion-exchangers have been detected in human brain (Havenga et al., 1994), and anion-exchange activity has been demonstrated in glial cells (Bourke et al., 1978) and in neurons (Hentschke et al., 2006). Although the functional role of acid-base disturbances in the brain is not comprehensively understood, disturbed acid-base transport in neurons and glia has been suggested to contribute to activation of glycolysis in astrocytes (Ruminot et al., 2011) and protect against depolarization-induced acidification in neurons (Svichar et al., 2011). Furthermore, a pathophysiological significance of acid-base transport in the brain has been demonstrated by studies linking acid-base transport function to altered excitability (Bell et al., 1999; Hentschke et al., 2006; Jacobs et al., 2008) and neuronal cytotoxicity (Cooper et al., 2009). It is likely that changes in both local intracellular and extracellular pH play a role for the effects of the acid-base transporters; and while the functional role of acid-base balance for control of autonomic nervous function is presently unclear, the prominent acid-base transport mechanisms demonstrated in brain regions putatively involved in cardiovascular control underscores the need for studies investigating their mechanistic importance.

Interestingly, it has been proposed that increased Na^+^ content in the body modifies sympathetic nervous activity and alters blood pressure due to a change in the concentration of Na^+^ in the cerebrospinal fluid (Blaustein et al., 2012). Transport of Na^+^ into or out of the cerebrospinal fluid takes place largely through the choroid plexus and is partly mediated by acid-base transporters, including NHE1, AE2, NCBE/NBCn2, NBCn1, and NBCe2 (Damkier et al., 2010) providing a novel putative mechanism linking acid-base transport to blood pressure control.

## Conclusions

Increasing evidence suggests that disturbed acid-base transport function may lead to clinically relevant changes in blood pressure and contribute to hypertension development. Although initial studies investigating the mechanistic link between acid-base transport and hypertension development are being conducted, we are still far from a comprehensive understanding. Additional studies are therefore required to determine to what extent altered acid-base transport function contributes to hypertension development in humans, whether these effects are due to changes in acid-base balance or dependent on the co- or counter-transported ions, which organ systems are primarily responsible for the changes in blood pressure, and whether the acid-base transport mechanisms can be exploited for future therapeutic intervention.

### Conflict of interest statement

The authors declare that the research was conducted in the absence of any commercial or financial relationships that could be construed as a potential conflict of interest.
